# Supercoiling Effects on Short-Range DNA Looping in *E*. *coli*

**DOI:** 10.1371/journal.pone.0165306

**Published:** 2016-10-26

**Authors:** Lauren S. Mogil, Nicole A. Becker, L. James Maher

**Affiliations:** 1 Department of Biochemistry and Molecular Biology, Mayo Clinic College of Medicine, 200 First St. SW, Rochester, Minnesota 55905, United States of America; 2 Biochemistry and Molecular Biology track, Mayo Graduate School, Mayo Clinic College of Medicine, 200 First St. SW, Rochester, Minnesota 55905, United States of America; University of California, Davis, UNITED STATES

## Abstract

DNA-protein loops can be essential for gene regulation. The *Escherichia coli lactose* (*lac*) operon is controlled by DNA-protein loops that have been studied for decades. Here we adapt this model to test the hypothesis that negative superhelical strain facilitates the formation of short-range (6–8 DNA turns) repression loops in *E*. *coli*. The natural negative superhelicity of *E*. *coli* DNA is regulated by the interplay of gyrase and topoisomerase enzymes, adding or removing negative supercoils, respectively. Here, we measured quantitatively DNA looping in three different *E*. *coli* strains characterized by different levels of global supercoiling: wild type, gyrase mutant (*gyrB226*), and topoisomerase mutant (*ΔtopA10*). DNA looping in each strain was measured by assaying repression of the endogenous *lac* operon, and repression of ten reporter constructs with DNA loop sizes between 70–85 base pairs. Our data are most simply interpreted as supporting the hypothesis that negative supercoiling facilitates gene repression by small DNA-protein loops in living bacteria.

## Introduction

DNA looping is a fundamental mechanism for the control of gene expression in prokaryotes [1, 2] and eukaryotes [3, 4]. DNA loops may span just a few turns of DNA, or many kilobase pairs, and are typically mediated by protein-DNA interactions. The *E*. *coli lac* operon provides a classic model for understanding control of gene expression by DNA looping [5–10] and for measuring DNA flexibility in vivo [11–14]. Lac repressor protein (LacI) is a tetramer that controls gene expression through its ability to simultaneously bind pairs of operator sites on DNA. The wild type *lac* operon contains three distinct operators with different LacI binding affinities. LacI binding to the proximal operator inhibits RNA polymerase binding to the *lac* promoter. Strikingly, 50–96% of the effective LacI concentration at the proximal operator is due to LacI bound at auxiliary operators colliding with the proximal operator via DNA looping [6, 14–19]. Besides protein competition, simply constraining a promoter in tightly-bent DNA is intrinsically repressive [12]. When allolactose or β-D-1-thiogalactopyranoside (IPTG) is present, these small molecules bind to allosteric sites on LacI and reduce its affinity for DNA, derepressing transcription of the *lac* operon.

Gene regulation and packaging of *E*. *coli* DNA take place in the context of negative supercoiling [20–23]. About half of total negative supercoiling in *E*. *coli* is constrained by DNA wrapping on proteins [24]. The remaining unconstrained supercoiling creates a high-energy, compacted plectonemic state. The extent of negative supercoiling changes as a function of cellular metabolic status and influences DNA replication and cell division [2, 24, 25]. The steady-state level of DNA negative supercoiling is maintained by the competing activities of DNA gyrase and topoisomerase enzymes. Gyrase (topoisomerase II) induces negative DNA supercoils by cutting both DNA strands and passing a DNA segment through this gap in an ATP dependent manner [26–30], increasing the negative linking number (*Lk*). Topoisomerases relieve negative supercoils through mechanisms involving transient cleavage of one or both DNA strands [27, 31, 32]. Mutations in gyrase and/or topoisomerase genes can alter regulation of supercoiling, resulting in perturbation of the steady-state level of negative supercoiling in *E*. *coli*. Such effects can also be achieved by pharmacological inhibitors of bacterial gyrase.

**Fig 1:**
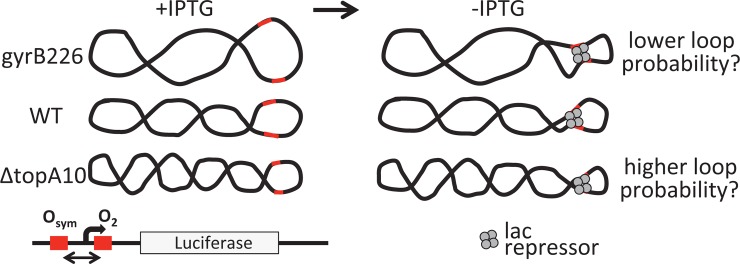
Experimental design for testing the hypothesis that short-range DNA looping by LacI is sensitive to negative superhelical density in vivo. Low, normal, and high negative superhelicities were achieved in *gyrB226*, WT (FW102 and JTT1), and Δ*topA10* genetic backgrounds, respectively. The firefly luciferase gene was inserted downstream of the *lac* UV5 promoter flanked by the indicated lac operators separated by 14 and 12 different spacings respectively. Reporter constructs were recombined onto single copy episomes encoding LacI. DNA loop stability is monitored as a function of operator spacing and IPTG induction.

Because supercoiling collapses DNA into interwound plectonemes, the process increases the local concentration of all DNA sites. It is therefore intuitive that supercoiling has the potential to increase the probability of operator bridging by proteins such as LacI, a tetrameric protein with two DNA binding sites that anchor loops between operators in the *lac* operon (Fig 1). Evidence of looping facilitation by supercoiling has been reported in some in vitro experiments [28, 33]. On the other hand, the measured local stiffness of DNA in vitro [18, 34–37] limits the extent to which global supercoiling can increase local operator concentrations for short DNA loops that may be relevant to *lac* control in vivo. Here we devised an approach to explicitly test the hypothesis that negative DNA supercoiling facilitates the formation of small (6–8 DNA turns) repression loops in *E*. *coli*.

Three bacterial strains [38, 39] are used in this study to test if the extent of negative supercoiling affects DNA looping. Strain JTT1 carries wild type copies of gyrase and topoisomerase genes and supports a negative supercoiling background that reflects normal *E*. *coli* cells. In contrast, strain KD112 carries partially defective gyrase mutation *gyrB226* so average negative superhelical density is more relaxed than in wild type cells. Strain RS2 carries partially defective topoisomerase mutant *ΔtopA10* resulting in increased negative DNA supercoiling relative to wild type cells. A second control strain, FW102, was used to study the current promoter-reporter constructs in the genetic background previously used by our laboratory [11, 40, 41].

Here we study control of the endogenous wild type *lac* operon and also apply an adaptation of the experimental system previously developed by Becker et al. to study DNA flexibility in living *E*. *coli* [11, 40, 41]. This system recapitulates aspects of previous classic designs created for this purpose [6, 15, 16, 19]. The present implementation places the firefly luciferase reporter gene downstream of a *lac* UV5 promoter flanked by a strong distal O_sym_ operator (upstream) and a weak proximal O_2_ operator (downstream). LacI saturates O_sym_ and increases the probability of O_2_ binding through looping of the intervening DNA. O_sym_ and O_2_ operator spacing is systematically varied in a series of constructs to measure the apparent bend and twist flexibilities of the intervening DNA. Promoter-reporter constructs are maintained in single copy by recombination onto an F’ episome [42]. Measurement of reporter repression as a function of operator spacing in the bacterial strain backgrounds differing in superhelical density allows a sensitive test of the hypothesis that negative supercoiling facilitates DNA looping in vivo (Fig 1). Classic studies and their analysis often involved comparing repression in bacteria possessing and lacking lac repressor [43–45]. This approach allows calculation of absolute repression. Here effects of supercoiling do not require assessment of absolute repression, so repression is expressed in relative terms using the behavior of a weakly repressed promoter with an isolated O_2_ operator as reference [11, 18].

## Materials and Methods

### Bacterial Strains

The bacterial strains used in this study are indicated in Table 1. Strain FW102 was used as a control to compare luciferase assay results to previous studies using β-galactosidase assays of the *lacZ* reporter gene [11]. *E*. *coli* K-12 derivatives JTT1 (wild type; WT) and isogenic mutant strains KD112 (*gyrB226*) and RS2 (Δ*topA10*) were generously provided by Dr. Peter Heisig [38].

**Table 1:** Bacterial strains used in this work.

Strain	Designation	Relevant genotype	Comment
FW102	WT	Strep^R^ derivative of CSH142. F-, *ara-600*, *Δ(gpt-lac)5*, LAM-, *relA1*, *spoT1*, *thiE1*	
JTT1	WT	F-, *gal-25*, λ-, *pyrF287*, *fnr-1*, *rpsL195(strR)*, *iclR7(Const)*, *trpR72(Am)*	Normal supercoiling
KD112	*gyrB226*	F-, *gal-25*, λ-, *pyrF287*, *fnr-1*, *rpsL195(strR)*, *iclR7(Const)*, *trpR72(Am)*, *gryB226*	Reduced negative supercoiling
RS2	*ΔtopA10*	F-, *gal-25*, λ-, topA10, *pyrF287*, *fnr-1*, *rpsL195(strR)*, *iclR7(Const)*, *trpR72(Am)*	Increased negative supercoiling

### Western blotting

Bacterial strains were cultured in 3 mL LB medium with appropriate antibiotics with aeration overnight at 37°C. Samples (100 μL) of each culture were then sub-cultured into 5 mL LB medium with appropriate antibiotics and grown to log phase (OD_600_ 0.5–0.7). Cells from 4.5 mL of culture were recovered by microcentrifugation at 13,500 rpm (Sorvall Legend Micro 17, Thermo Scientific) for 2 min and resuspended in 200 μL 1× MES buffer (50 mM MES, 50 mM Tris-HCl, 3 mM SDS, 1 mM EDTA, pH 7.5). Cells were lysed by sonication in bursts of 10 s and cooled on ice for 30 s. Samples were clarified by microcentrifugation and protein content quantitated using a BCA kit (Pierce) [46]. Samples were analyzed by electrophoresis through 10% bis-Tris polyacrylamide gels at 150 V for 1.5 h using 10 μg total protein for anti-LacI (1:10,000; LSBio) and anti-σ^70^ (1:10,000; NeoClone) antibodies or 5 μg total protein for anti-RNAPα (1:15,000, NeoClone) antibody and an Αn anti-HUβ polyclonal antiserum (5 μg; 1:10,000) was prepared by immunization of rabbits with peptide conjugates and subsequent affinity purification. Protein was transferred to PVDF membrane using NuPAGE transfer buffer (Thermo Fisher Scientific) [47] containing 20% methanol. Transfer conditions were 30 V for 90 min at 4°C. Blots were blocked overnight in 5% dry milk in 1X TBST buffer and incubated with primary antibody for 2 h followed by secondary HRP-conjugated anti-mouse/rabbit antibody (1:30,000; Promega) for 1 h. ECL plus kit (Pierce) was used to detect the protein, exposed to x-ray film, and imaged by photographic scanning.

### Chloroquine gel analysis of DNA supercoiling

WT, *gyrB226*, and *ΔtopA10* strains were transformed with plasmid pUC19 derivative pJ1506 and grown to log phase (OD_600_ 0.5–0.7) in 250 mL LB medium with appropriate antibiotics. Plasmids were purified (Qiagen Maxi prep kit) with care to minimize nicking. DNA concentration was determined by UV spectrophotometry using a Nanodrop 1000 (Thermo scientific). Vertical electrophoresis was performed between glass plates (21.5 cm X 20.5 cm) separated by a 1-mm spacer. 1.4% agarose gels contained the indicated concentrations of chloroquine (made up from 1 mg/mL stock; Sigma). Electrophoresis was in 1× TAE buffer (40 mM Tris acetate, 1 mM EDTA) at 4 V/cm for 15 h. The electrophoresis buffer (800 mL) was recirculated at a rate of 55 mL/h. Gels were then incubated for 1 h in electrophoresis buffer containing ethidium bromide (0.5 μg/mL, Sigma) and destained in water for 30 min. Images were recorded on a Typhoon fluorescence imager (FLA7000 GE) and analyzed using ImageJ software. Quantitation of DNA supercoiling as linking number deficit was accomplished using the band counting method as described [31, 48]:

$$\sigma =\Delta Lk/Lk_{o}$$ (1)

where *Lk*_0_ is the linking number of the relaxed plasmid [number of base pairs (2,700) divided by the helical repeat (10.5 bp/turn)]. *ΔLk* is the change in linking number, determined by band counting from the position of the relaxed plasmid (intact but not supercoiled) band (just below the nicked marker at the top of the gel) to the most abundant topoisomer band for each chloroquine concentration, and plotting the relationship between most abundant topoisomer and chloroquine concentration. Care was taken to confine the analysis to chloroquine concentration conditions where a minimal fraction of topoisomers had been driven to a positively supercoiled state. The extrapolated y-intercept value at zero chloroquine (rounded to the nearest integer) is the estimate of *ΔLk*.

### Looping constructs

DNA looping constructs were based on plasmid pJ992, as previously described [11]. Constructs contain a strong upstream O_sym_ operator and a weak proximal O_2_ operator flanking a lac UV5 promoter. A construct with the proximal O_2_ operator but lacking O_sym_ was used as a normalization control [11]. The firefly luciferase reporter gene was amplified by PCR from plasmid pJ1454 using primers LJM-5191 5’CGA_2_T_3_CGAC_2_TGCA_2_TG_2_A_2_GACGC_2_A_5_C and LJM-5192 5’GC_2_A_2_GCT_2_G_2_CTGCAT_2_AT_2_ACA_2_T_3_G_2_ACT_3_C_2_G and placed upstream of an existing *lacZ* reporter gene in the tested series of plasmids using a Gibson assembly kit (NEB) [49]. Plasmids containing the indicated center-to-center operator spacings (S3 Table) were digested with *Pst*I (NEB) prior to Gibson assembly. Plasmid sequences were verified by Sanger sequencing using primer LJM-1925 5’ T_2_CA_3_TATGTATC_2_GCTC.

### Bacterial conjugation for episome transfer

Assembled plasmids were transformed into the BL536 donor strain containing a single copy F128 episome [42]. Constructs were moved to this F' episome by homologous recombination. The F128 episome encodes a *LacI* gene producing wild-type levels of repressor. The WT, gyrB226, and Δ*topA10* strains contain an endogenous chromosomal copy of *lac* operon, leading to increased concentrations of Lac repressor. Bacterial conjugation and selection for recombinant F' episomes was performed as described [42].

### β-Galactosidase assays for native *lac* operon

Test strains were grown overnight in M9 minimal medium (1X M9 salts, 5mM MgSO_4_, 0.2% Casamino acids, 0.01% thiamine, 1mM CaCl_2_) supplemented with 20 mM uracil and 0.8% glycerol. Strains were then subcultured into 4 mL of minimal media with and without 2 mM IPTG and grown to OD_600_ ~0.3 in deep-well culture boxes. Samples (100 μL) were diluted into 900 μL Z buffer (60mM Na_2_HPO_4_, 40mM NaH_2_PO_4_, 10 mM KCl, 1 mM MgSO_4_, 50 mM β-mercaptoethanol) and lysed by addition of 50 μL chloroform and 25 μL 0.1% SDS followed by vortex mixing. Samples were incubated at 30°C for five min and 200 μL O-nitrophenyl-pyrano-galactoside (ONPG; 4 mg/mL) was added. Reactions were stopped by adding 500 μL 1M Na_2_CO_3_ when an appropriate range of yellow product color was achieved. The reaction time was recorded. Cell debris was pelleted by centrifugation using a microcentrifuge at 13,500 rpm. Sample optical density (OD) readings were recorded at wavelengths of 420 and 550 nm. β-Galactosidase activity was calculated according to:

$$E=1000\frac{(OD_{420}-1.75\left(OD_{550}\right)}{t*v*OD_{600}}$$ (2)

where OD_x_ refers to the optical density at the indicated wavelength, *t* is the reaction time in minutes, and *v* indicates culture volume in mL [50]. Assays were performed for cultures derived from at least six colonies from each independent strain and repeated on three different days.

### Luciferase reporter assays

Bacterial cultures (1 mL) were grown in LB broth with appropriate antibiotics using deep-well culture boxes shaking at 37°C overnight. Subcultures (50 μL) were inoculated into 1 mL LB media with appropriate antibiotics and grown to log phase (OD_600_ 0.5–0.7) with and without 2 mM IPTG. Once at log phase, samples (90 μL) were supplemented with 10 μL buffer (1M K_2_HPO_4_ and 20 mM EDTA) and frozen at -80°C for 30 min. Samples were then thawed at room temperature and incubated with 200 μL 1× CCLR buffer (Promega) together with 100 μL of solution containing lysozyme (5 mg/ mL; Sigma) and BSA (5 mg/mL; Sigma) for 10 min. Lysate samples of precise volume (10–20 μL) were analyzed (Promega GloMax^TM^ 96 microplate luminometer) with automated injection of 50 μL luciferase assay reagent solution (Promega). Each sample was read in duplicate. The luciferase activity was calculated according to:

$$E=\frac{\left(LU/v\right)}{OD_{600}}$$ (3)

where *LU* are luminometer light units, *v* is the sample volume analyzed in microliters, and *OD*_600_ is the sample optical density at 600 nm. Assays were performed for cultures derived from at least six colonies from each independent strain and repeated on three different days.

### DNA looping analysis

The repression ratio (*RR*) is given by the ratio of the raw induced luciferase activity divided by the repressed luciferase activity:

$$RR=\frac{E_{+IPTG}}{E_{-IPTG}}$$ (4)

The repression level, *RL*, is given by expression from the completely induced promoter in the absence of looping to the completely repressed promoter in the presence of looping

$$RL=\frac{E_{O_{2}+IPTG}}{E_{O_{sym}O_{2}–IPTG}}$$ (5)

While it is possible to extract fits to parameters related to DNA physical properties from repression data [11, 16, 18, 51–53], the present analysis of supercoiling effects on repression is presented qualitatively.

## Results and Discussion

### Characterizing DNA supercoiling in experimental bacterial strains

The three bacterial strains studied here were selected because they have been previously shown to sustain different levels of negative superhelical density during log phase growth [26, 28, 39]. We confirmed these differences in negative superhelical density using two assays.

First, a qualitative luciferase reporter assay [54] was performed to measure unrestrained negative supercoiling. This assay involves transformation of bacteria with plasmids encoding luciferase reporter genes driven by *gyr* or *topo* promoters, which are differentially sensitive to negative superhelical strain. The activity of each promoter was measured by its corresponding luciferase activity and quotients of the activities are reported in Table 2. These results are similar to a previous report [54] and confirm that the gyrB226 and Δ*topA10* strains have decreased and increased unrestrained negative superhelical density, respectively, relative to WT.

**Table 2:** Confirmation of supercoiling status by torsion-sensitive promoter assay^a^.

Designation	*E*_*topo*_*/E*_*gyr*_	previously reported value [54]
WT	4.33±0.107	2.67
*gyrB226*	1.40±0.022	1.10
*ΔtopA10*	9.45±0.346	5.29

^a^Ratio of luciferase activities driven by the indicated promoters (mean ± standard deviation n = 9 for each strain)

**Fig 2:**
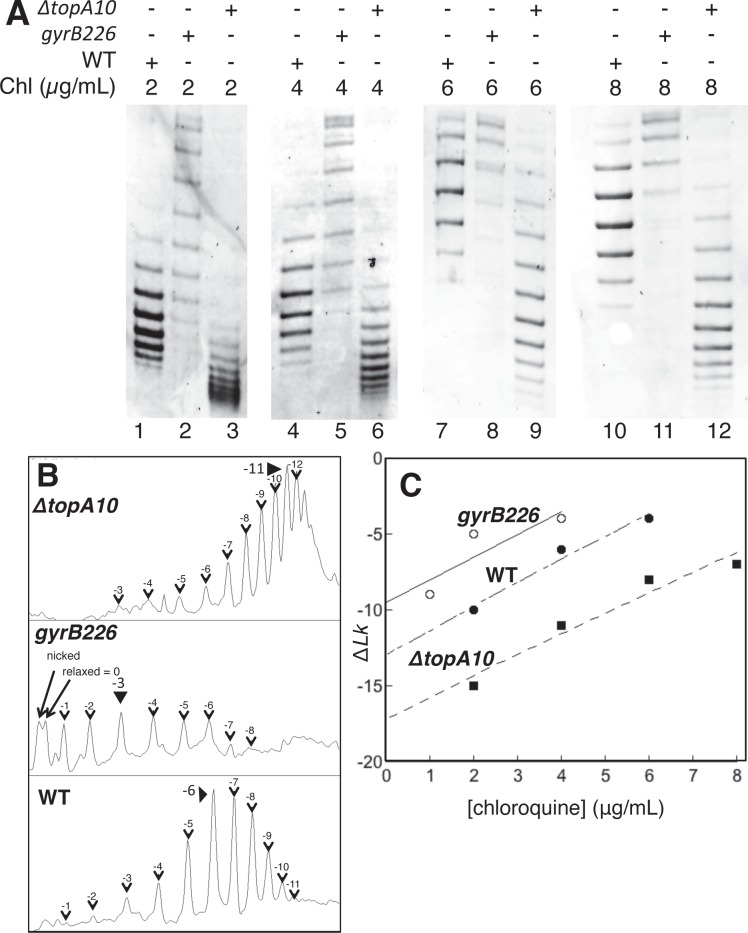
Confirmation of different negative superhelical densities in experimental bacterial strains. A plasmid pUC19 derivative was transformed into the indicated strains. A. Bacteria were grown to log phase and extracted plasmids electrophoresed through gels containing the indicated concentrations of chloroquine to separate topoisomers. B. Topoisomer distributions were evaluated by band counting and densitometry (4 μg/mL) to identify the most prevalent topoisomer for each strain. C. Most abundant topoisomer was plotted as a function of chloroquine concentration allowing extrapolation to zero chloroquine to establish plasmid superhelical density *in vivo* (Materials and Methods).

We then further quantitated total negative superhelical density in each strain using chloroquine gel analysis. The topopoisomer distribution for plasmids isolated from each strain was detected after agarose gel electrophoresis in the presence of increasing concentrations of chloroquine, an intercalator that relaxes negative supercoils and eventually adds positive supercoils (Fig 2A). Lanes 1, 4, 7, and 10 of Fig 2A show plasmid DNA isolated from the WT strain electrophoresed in the presence of different concentrations of chloroquine where negative topoisomers predominate. As the chloroquine concentration increases, negative supercoils in the plasmid unwind and migration through the gel is retarded. Plasmids isolated from *gyrB226* mutant cells (Fig 2A; lanes 2, 5, 8, 11) are less supercoiled than WT. Plasmids isolated from *ΔtopA10* cells (Fig 2A; lanes 3, 6, 9, 12) are more negatively supercoiled. After identifying the most prevalent topoisomer (Fig 2B) the supercoiling density (σ) was calculated for each strain by the band counting method [31, 48]. Increasingly supercoiled topoisomers are counted as bands of increasing mobility relative to the relaxed position (closest to nicked DNA control). Care was taken to assign the most abundant topoisomer band in chloroquine concentrations where negatively supercoiled states predominated and a minimal fraction of topoisomers had been driven to positively supercoiled states by chloroquine binding. Extrapolation to zero chloroquine using the *y*-intercept as *ΔLk* (Fig 2C) yielded σ values of -0.05 (WT), -0.04 (*gyrB226*) and -0.07 (*ΔtopA10*) as calculated from Eq 1 (Materials and Methods). The observed value for σ in WT cells is comparable to a prior report [55]. We conclude from these studies that the experimental strains do support significantly different negative superhelical densities during log phase growth, as expected.

### Regulatory protein expression in experimental bacterial strains

**Fig 3:**
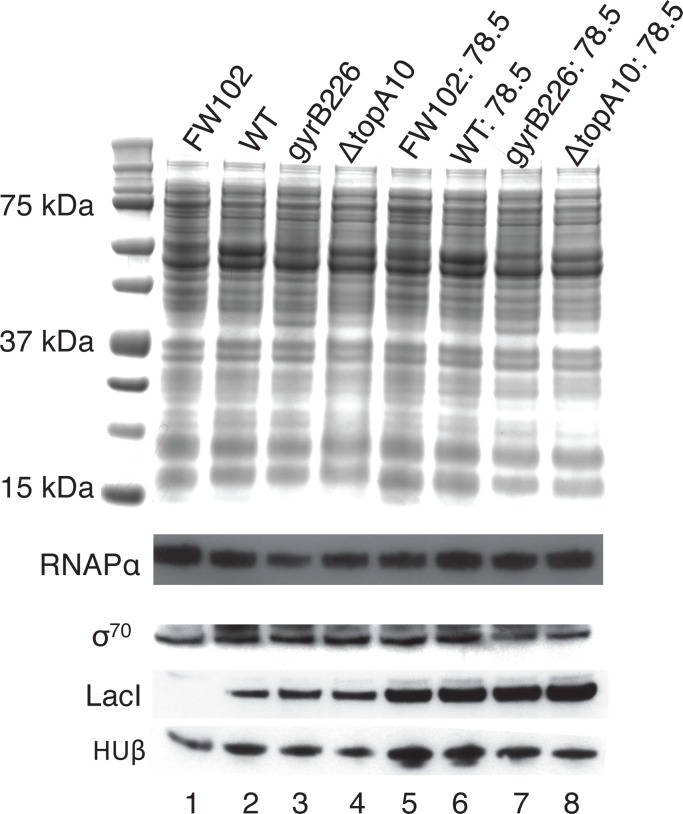
Protein expression in experimental bacterial strains. Proteins from the indicated strains were extracted at log phase and analyzed by coomassie staining (above), or by western blotting (below) with antibodies specific for the indicated proteins.

Our approach to measuring effects of negative superhelical strain on short-range DNA looping exploits components of the *lac* control region to assess DNA loop stability. Comparison between strains requires an understanding of if and how the strains differ in the concentrations of proteins important for DNA looping, such as HU, IHF, Fis, H-NS, and LacI. Differential expression of such proteins could alter DNA looping for indirect reasons unrelated to superhelical strain within the looping region [21]. Expression levels of other regulatory proteins that control overall levels of *lac* gene expression (e.g. RNA polymerase, sigma factor) are also of interest. A previous comprehensive report [25] demonstrated that changes in supercoiling did not alter expression of LacI, HU, IHF, Fis, H-NS or RNA polymerase. Expression of the σ^70^ protein was reportedly induced by relaxing supercoils. To confirm aspects of this prior report, the protein expression status of the relevant strains was analyzed by western blotting (Fig 3). As previously reported, total protein expression (Fig 3, top) as well as expression of RNA polymerase alpha subunit (RNAPα) and HU β subunit (Fig 3, bottom) was confirmed not to differ greatly between strains. HU β subunit was slightly reduced in the Δ*topA10* mutant (see below) and the possible induction of σ^70^ was not detected in the *gyrB226* mutant. It is important to note that these results cannot exclude the possibility that other differences of gene expression could contribute to indirect effects on DNA looping differences between strains. Blotting for LacI confirmed that the FW102 strain lacks LacI expression, as expected (Fig 3, lane 1), and that equal levels of LacI are detected in FW102 extracts when the *LacI* gene is present in the F' episome (Fig 3, lanes 2–4). Equivalent (higher) levels of LacI are detected, as expected, in experimental strains containing *LacI* gene copies in both the chromosome and the F' episome (Fig 3, lanes 5 vs. 6–8). Importantly, our experiments only make DNA looping comparisons between strains with comparable LacI expression levels. We conclude that the relevant experimental strains (Fig 3, lanes 6–8) are comparable in expression of relevant gene regulatory proteins important for DNA looping assays.

### DNA looping in the endogenous *lac* operon as a function of supercoiling

**Fig 4:**
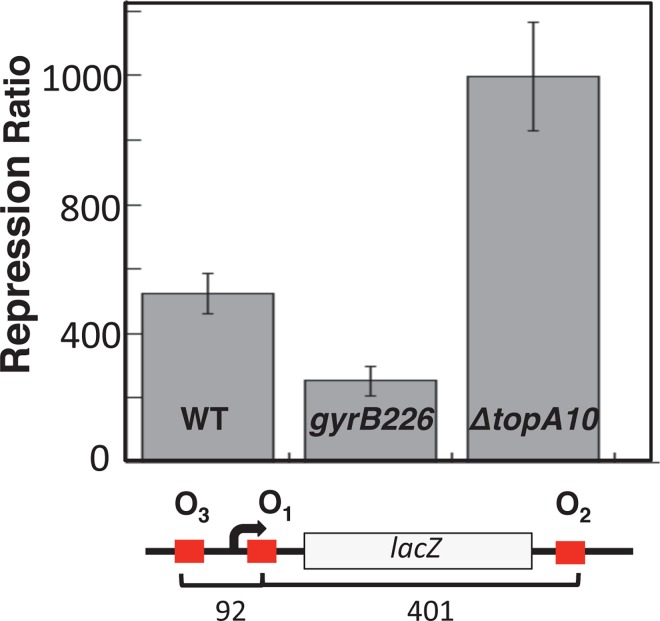
β-galactosidase assay results for control of the endogenous *lac* operon (schematic) in the indicated assay strains. Standard deviations are based on at least 6 measurements.

The WT, *gyrB226*, and *ΔtopA10* strains each contain an endogenous chromosomal copy of the *lac* operon whose repression depends on DNA looping. Before testing looping as a function of operator spacing in engineered F' episome constructs, initial β-galactosidase assays were performed to assess control of the endogenous *lac* operon as a function of negative supercoiling. Experiments were done in minimal medium supplemented with glycerol in order to engage the catabolite activator protein for maximal *lac* promoter induction. The repression ratio (β-galactosidase activity in the presence vs. absence of IPTG) was calculated for each strain (Fig 4). Repression ratio values followed the ranking *ΔtopA10* > WT > *gyrB226* (Fig 4). Because *lac* repression is primarily due to DNA looping [19], this result supports the hypothesis that global negative supercoiling promotes DNA repression loop formation in vivo.

### DNA looping as a function of loop length and superhelical density

The endogenous *lac* operon is controlled by three potential loops of fixed lengths. To assess repression by a single control loop over a range of loop lengths in these experimental strains, looping constructs based on a luciferase reporter gene were created to provide a readout independent from the endogenous *lac* operon in the test strains.

**Fig 5:**
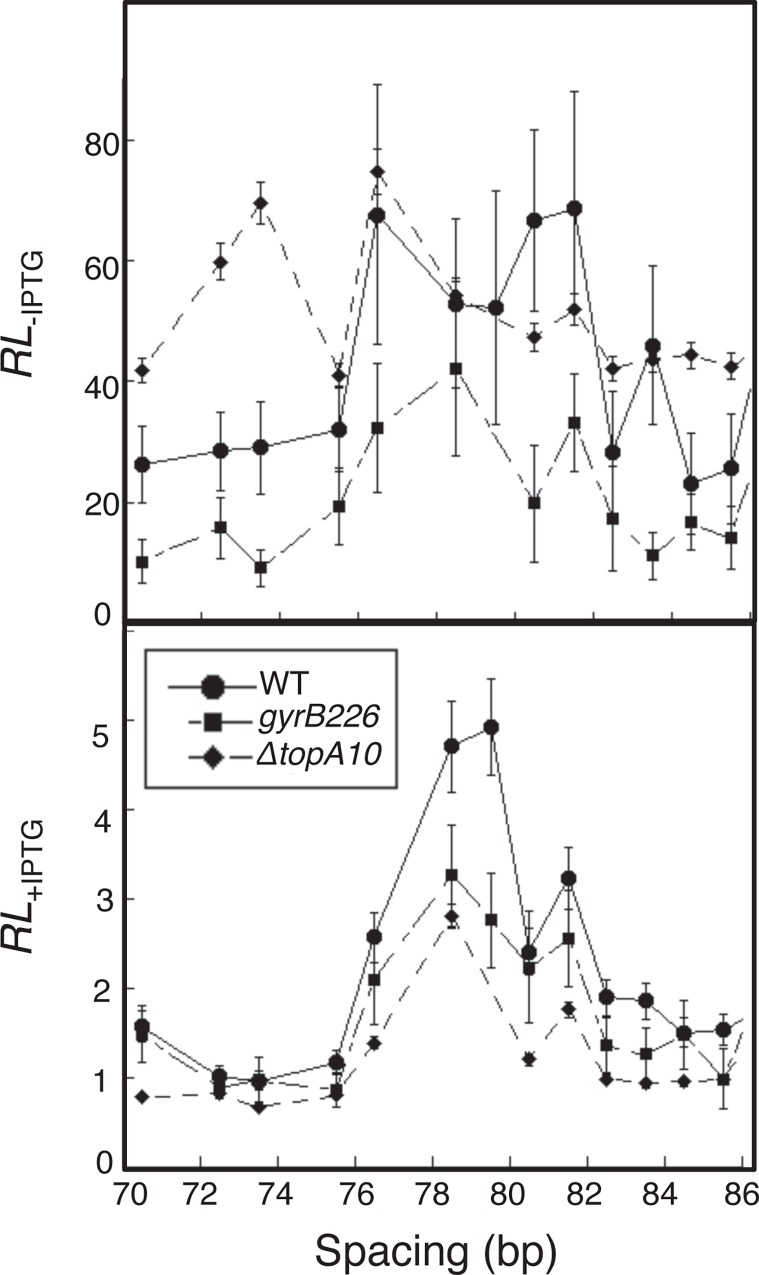
Repression level (*RL*, defined in Eq 5) as a function of operator spacing and DNA negative superhelical density is shown as a function of operator spacing for the indicated bacterial strains. Upper and lower panels are data collected in the absence or presence of IPTG, respectively. Propagated error estimates are based on data from at least 6 measurements.

Assessing looping across a range of operator spacings surrounding a strong engineered *lac* UV5 promoter is important to appreciate repression at the optimal operator spacing for each supercoiling condition. Luciferase activity was assayed in the absence and presence of IPTG induction during log phase growth for each strain (S1 Table). Reporter expression (*E'*; expression normalized to a construct incapable of looping) or repression level (*RL*; expression comparing fully induced state to fully repressed state) were measured as a function of operator separation. The results are shown in Fig 5.

The results of these studies confirm the previous observation [11, 40, 41] that repression oscillates with the separation of *lac* operators, such that tightest repression occurs when torsionally relaxed DNA loops can form (~78.5 bp center-to-center operator separation), and repression is weakest when loops are twisted. This oscillation pattern is particularly evident in the case of residual DNA looping in the presence of IPTG [11] as seen for all three test strains in the lower panel of Fig 5.

With respect to DNA loop stability over 6–8 turns of DNA, the impact of negative superhelical density is most evident from the upper panel of Fig 5, where repression of gene expression is compared as a function of operator spacing for the three test strains. The supercoiling-deficient *gyrB226* strain exhibits the leakiest behavior, also consistent with the hypothesis being tested. Under these conditions repression in the WT strain is intermediate for most spacings, and repression in the *ΔtopA10* strains is superior for most operator spacings. Interestingly, the pattern of spacing-dependent repression oscillation varies among strains in the absence of IPTG. The hyper-supercoiled *ΔtopA10* strain displays strong repression levels most broadly without loop length-dependence. One interpretation of this result is that strong negative supercoiling decreases the barrier of DNA twisting to repression loop formation. In summary, these data show that in the cases where there are differences in apparent LacI repression loop stability between hosts, these differences are in the order *ΔtopA10* > WT > *gyrB226*, mirroring the ranking of host negative superhelical density. While we cannot completely rule out the possibility that strain differences other than superhelical density effects on the DNA loop influence these results, the simplest interpretation of these data is that short-range DNA looping is facilitated by negative DNA supercoiling in vivo.

Interestingly, where optimal operator spacing can be detected in Fig 5, it does not appear to be strongly dependent on the supercoiling level in the experimental strain. Apparently over highly-constrained and short-range loops such as these (~7 turns of DNA), superhelical strain increases local operator concentration without large changes in optimal repressor spacing. This suggests that the helical repeat of the DNA is not largely changed between these three test strains.

### Experimental considerations in vivo

Here we studied effects of DNA supercoiling on DNA looping using bacterial strains carrying mutations that alter global supercoiling. We adopted this approach after initially considering control of supercoiling using antibiotics. For example, the use of fluoroquinolone (FQ) antibiotic inhibitors of DNA gyrase has been used historically to relax negative superhelical density in *E*. *coli* [26, 38, 56]. We found that FQ addition rapidly arrested cell growth, making it difficult to analyze gene expression under conditions of log-phase growth important to allow comparison between strains. More importantly, convenient reporter enzymes have sufficiently long lifetimes that a significant fraction of measured reporter activity would reflect molecules synthesized prior to FQ addition, confounding analysis.

Likewise, because of the relationship between culture density and supercoiling status [1, 2] we were cautious to assess *lac* control during log phase growth at a comparable growth density for each strain after confirming that all three strains reach the same degree of culture saturation at long growth times.

We caution that comparison of DNA looping strength by monitoring operator spacing dependence of repression is subject to the validity of the assumption that looping regulatory protein expression (e.g. LacI, HU, IHF, Fis, H-NS) is not different between test strains. There is prior support for this assumption [25] and our western blotting experiments tend to validate it. Note that the slight decrease in HUβ protein detected in the *ΔtopA10* strain cannot account for the improved repression observed in that strain. If HU is important for DNA looping as suggested by prior work [11] the slight reduction in HUβ expression would actually tend to *decrease* looping and repression. Thus, the HUβ expression change might actually be masking an even larger repression improvement due to increased negative supercoiling in the *ΔtopA10* strain. No differences in RNA polymerase or σ^70^ levels were observed between strains, and any such differences would have been controlled by the *E'* reporter expression parameter, which is normalized for the expression of a reporter controlled by a single *lac* operator not subject to looping. Test strains with different levels of LacI expression were created. Control constructs with single weak O_2_ operators and no looping confirmed that doubling the *laci* gene copy number from one to two per cell approximately doubled basal repression, as expected. We emphasize that direct comparisons always involved strains expressing the same level of LacI.

### Supercoiling effects on large and small DNA loops

Several groups have explored the relationship between supercoiling and DNA looping in vitro [13, 28, 33, 57–59]. Interesting recent experiments suggest that *lac* repressor can constrain supercoils within the large (401-bp) looped domain of the native *lac* operon (not studied here), suggesting that supercoiling can enhance looping and repression of the *lac* promoter [60]. Likewise, negative supercoiling was known to stabilize loops and subtle changes in supercoiling changed the optimal operator spacings for LacI looping in DNA minicircles *in vitro* [14, 33].

Our results extend prior work by documenting in vivo that negative supercoiling promotes the formation of even small (65–86 bp) loops. Because global DNA supercoiling is dependent on energy status and cell growth phase, our results suggest that genes whose expression is regulated by DNA loops may be induced or repressed as a function of this global DNA supercoiling status. It is interesting to conjecture that the effect of negative supercoiling on DNA looping may be loop-length dependent. For DNA lengths lower than one persistence length, resistance to looping is expected to be dominated by local DNA bending and twisting energies. Under these conditions, extensive DNA bending may be particularly facilitated by energy stored in superhelical strain. Comparing facilitation of small and large DNA loops by negative supercoiling will be interesting [60, 61]. Perhaps future Monte Carlo simulations, coupled with experiments, will allow this supercoiling-looping relationship to be understood in vitro and in vivo.

## Supporting Information

S1 Fig Verification of luciferase reporter method. FW102 bacterial strains (Table 1) used in previous Becker et al. looping studies with same F’ episomes transferred as WT and measured for activity. Both strains exhibit similar luciferase activity indicating that looping can be adequately measured using this new reporter method. The difference in the uninduced measurements could be attributed to the native *lac* operon and lac repressor in WT strain that is lacking in the FW102 strain.
(PDF)

S1 Table Raw *E* data. (DOCX)

S2 Table Repression Ratios. (DOCX)

S3 Table Looping constructs, operator spacings, and strain designations. (DOCX)
