# Supplementary material for: Supercoiling Effects on Short-Range DNA Looping in E. coli
Source: PLoS One. 2016 Oct 26;11(10):e0165306. doi: 10.1371/journal.pone.0165306 (PMC5081198; doi:10.1371/journal.pone.0165306)
Supplement: S1 Fig — FW102 bacterial strains (Table 1) used in previous Becker et al. looping studies with same F’ episomes transferred as WT and measured for activity. Both strains exhibit similar luciferase activity indicating that looping can be adequately measured using this new reporter method. The difference in the uninduced measurements could be attributed to the native lac operon and lac repressor in WT strain that is lacking in the FW102 strain. (PDF) [file pone.0165306.s001.pdf]

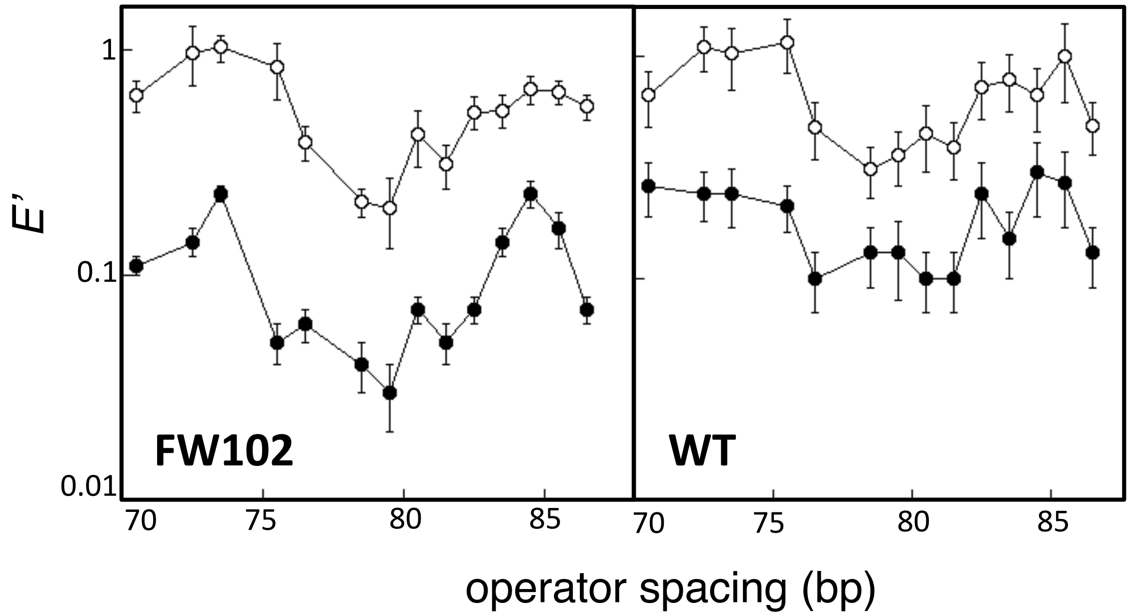

S1 Figure. Validation of luciferase reporter. E' is E for the test looping construct divided by E for a construct carrying only O2 in the proximal position.
