# Supplementary material for: Supercoiling Effects on Short-Range DNA Looping in E. coli
Source: PLoS One. 2016 Oct 26;11(10):e0165306. doi: 10.1371/journal.pone.0165306 (PMC5081198; doi:10.1371/journal.pone.0165306)
Supplement: S1 Table — (DOCX) [file pone.0165306.s002.docx]

**S1 Table**. Raw *E* data

| Spacing | FW Ave -IPTG | FW Ave +IPTG | WT Ave -IPTG | WT Ave +IPTG | gyrB226 Ave -IPTG | gyrB226 Ave +IPTG | *Δ*topA10 Ave -IPTG | *Δ*topA10 Ave +IPTG |
| --- | --- | --- | --- | --- | --- | --- | --- | --- |
| 70.5 | 1631.10±  87.88 | 31234.77±  87.88 | 1354.43±  156.31 | 23939.35±  4440.96 | 1822.29±  486.12 | 23861.44±  4047.73 | 536.45±  123.72 | 9349.94±  1602.19 |
| 72.5 | 2078.23±  192.46 | 48521.48±  192.46 | 1249.11±  106.80 | 39481.07±  4120.58 | 1186.91±  278.73 | 22548.99±  3895.78 | 375.63±  52.18 | 14925.47±  2589.74 |
| 73.5 | 3282.48±  198.11 | 50766.86±  198.11 | 1226.48±  182.21 | 36516.86±  8437.53 | 2031.59±  518.86 | 27514.91±  4258.44 | 323.11±  43.09 | 19513.99±  3178.76 |
| 75.5 | 777.32±  50.33 | 41695.15±  50.33 | 1106.97±  51.34 | 41280.55±  7359.11 | 970.08±  246.84 | 22943.21±  6296.05 | 547.79±  46.59 | 22623.37±  1320.38 |
| 76.5 | 860.37±  149.15 | 19175.96±  149.15 | 526.12±  125.50 | 17009.20±  3488.60 | 577.07±  146.45 | 13459.86±  2527.72 | 300.04±  39.40 | 9078.58±  1701.37 |
| 78.5 | 570.94±  67.58 | 10491.43±  67.58 | 673.20±  106.66 | 10888.01±  1476.16 | 443.89±  119.55 | 6658.04±  1746.16 | 413.24±  52.72 | 5007.41±  840.78 |
| 79.5 | 438.91±  129.86 | 10030.77±  129.86 | 681.00±  206.41 | 12867.76±  2039.98 |  |  |  |  |
| 80.5 | 981.44±  187.54 | 20480.48±  187.54 | 533.80±  42.06 | 15881.55±  4055.13 | 942.95±  418.23 | 15507.60±  8467.71 | 475.06±  81.04 | 6295.60±  990.32 |
| 81.5 | 740.97±  130.10 | 15272.18±  130.10 | 517.75±  95.14 | 13950.21±  2520.34 | 561.76±  65.67 | 10593.24±  2707.19 | 433.48±  47.18 | 5716.80±  383.04 |
| 82.5 | 1006.42±  155.67 | 26035.50±  155.67 | 1262.03±  361.70 | 26066.50±  5218.01 | 1080.04±  498.18 | 18923.48±  6360.21 | 533.18±  97.54 | 9723.22±  2946.07 |
| 83.5 | 2044.05±  195.03 | 26553.73±  195.03 | 773.59±  146.01 | 28056.61±  5831.73 | 1679.77±  485.80 | 19909.83±  3496.49 | 514.00±  78.80 | 12418.50±  1592.34 |
| 84.5 | 3268.54±  333.01 | 32849.91±  333.01 | 1543.51±  449.98 | 24013.61±  5598.50 | 1121.74±  198.02 | 19440.44±  3538.75 | 506.55±  125.64 | 11909.32±  3146.19 |
| 85.5 | 2338.64±  463.59 | 32097.16±  463.59 | 1391.20±  389.99 | 35967.00±  11408.45 | 1318.34±  386.19 | 19061.48±  6228.77 | 528.51±  85.70 | 15693.19±  2373.77 |
| 86.5 | 1049.48±  126.05 | 27776.88±  126.05 | 690.15±  133.13 | 17405.60±  3019.22 | 543.26±  38.69 | 11654.85±  985.66 | 478.64±  45.23 | 14846.94±  1130.32 |
| O_2_ alone | 14374.63+ 983.44 | 49345.3+ 5361.18 | 5162.98+ 1211.1 | 35579.19+7540.37 | 2379.25+ 518.57 | 18682.28+3937.78 | 2209.92+ 583.83 | 22458.86+ 5594.68 |
|  |  |  |  |  |  |  |  |  |
