# Supplementary material for: Supercoiling Effects on Short-Range DNA Looping in E. coli
Source: PLoS One. 2016 Oct 26;11(10):e0165306. doi: 10.1371/journal.pone.0165306 (PMC5081198; doi:10.1371/journal.pone.0165306)
Supplement: S2 Table — (DOCX) [file pone.0165306.s003.docx]

**S2 Table**. Repression level (*RL*; O_2_ alone +IPTG/O_sym_-O_2_ -IPTG)

| Spacing | FW102 | WT | gyrB226 | *Δ*topA10 |
| --- | --- | --- | --- | --- |
| (bp) |  |  |  |  |
| 70.5 | 30.25± | 26.27± | 10.25± | 41.87± |
|  | 3.67 | 6.34 | 3.49 | 14.21 |
| 72.5 | 23.74± | 28.48± | 15.74± | 59.79± |
|  | 3.39 | 6.51 | 4.97 | 17.05 |
| 73.5 | 15.03± | 29.01± | 9.20± | 69.51± |
|  | 1.87 | 7.51 | 3.05 | 19.64 |
| 75.5 | 63.48± | 32.14± | 19.26± | 41.00± |
|  | 8.03 | 6.97 | 6.36 | 10.79 |
| 76.5 | 57.35± | 67.63± | 32.37± | 74.85± |
|  | 11.73 | 21.58 | 10.68 | 21.08 |
| 78.5 | 86.43± | 52.85± | 42.09± | 54.35± |
|  | 13.89 | 13.98 | 14.39 | 15.21 |
| 79.5 | 112.43± | 52.25± |  |  |
|  | 35.44 | 19.32 |  |  |
| 80.5 | 50.28± | 66.65± | 19.81± | 47.28± |
|  | 11.05 | 15.07 | 9.73 | 14.27 |
| 81.5 | 66.60± | 68.72± | 33.26± | 51.81± |
|  | 13.75 | 19.28 | 8.02 | 14.08 |
| 82.5 | 49.03± | 28.19± | 17.30± | 42.12± |
|  | 9.27 | 10.05 | 8.77 | 13.02 |
| 83.5 | 24.14± | 45.99± | 11.12± | 43.69± |
|  | 3.49 | 13.05 | 3.98 | 12.78 |
| 84.5 | 15.10± | 23.05± | 16.65± | 44.34± |
|  | 2.25 | 8.31 | 4.58 | 15.59 |
| 85.5 | 21.10± | 25.57± | 14.17± | 42.49± |
|  | 4.77 | 8.99 | 5.11 | 12.63 |
| 86.5 | 47.02± | 51.55± | 34.39± | 46.92± |
|  | 7.61 | 14.77 | 7.65 | 12.5 |
|  |  |  |  |  |
