# Supplementary material for: Supercoiling Effects on Short-Range DNA Looping in E. coli
Source: PLoS One. 2016 Oct 26;11(10):e0165306. doi: 10.1371/journal.pone.0165306 (PMC5081198; doi:10.1371/journal.pone.0165306)
Supplement: S3 Table — (DOCX) [file pone.0165306.s004.docx]

**S3 Table.** Looping constructs, operator spacings, and strain designations

| Spacing | plasmid | FW102 (BL383) | WT (BL1191) | *gyrB226* (BL1198) | *ΔtopA* (BL1199) |
| --- | --- | --- | --- | --- | --- |
|  |  | strain designation | | | |
| 70.5 | pJ2248 | BL1215 | BL1225 | BL1236 | BL1246 |
| 72.5 | pJ2249 | BL1216 | BL1226 | BL1237 | BL1247 |
| 73.5 | pJ2250 | BL1217 | BL1227 | BL1238 | BL1248 |
| 75.5 | pJ2251 | BL1218 | BL1228 | BL1239 | BL1249 |
| 76.5 | pJ2252 | BL1219 | BL1229 | BL1240 | BL1250 |
| 78.5 | pJ2253 | BL1202 | BL1204 | BL1208 | BL1209 |
| 79.5 | pJ2254 | BL1257 | BL1260 | BL1263 | BL1266 |
| 80.5 | pJ2255 | BL1258 | BL1261 | BL1264 | BL1267 |
| 81.5 | pJ2256 | BL1259 | BL1262 | BL1265 | BL1268 |
| 82.5 | pJ2257 | BL1220 | BL1230 | BL1241 | BL1251 |
| 83.5 | pJ2258 | BL1221 | BL1231 | BL1242 | BL1252 |
| 84.5 | pJ2259 | BL1222 | BL1232 | BL1243 | BL1253 |
| 85.5 | pJ2260 | BL1223 | BL1233 | BL1244 | BL1254 |
| 86.5 | pJ2261 | BL1224 | BL1234 | BL1245 | BL1255 |
| O_2_ alone | pJ2262 | BL1200 | BL1235 | BL1206 | BL1256 |
